# Chemokine gradients spare graft endothelium from CD8^+^ T cell–mediated injury during allograft rejection

**DOI:** 10.1172/JCI193454

**Published:** 2025-07-15

**Authors:** Scott M. Krummey, Jonathan S. Bromberg

**Affiliations:** 1 Department of Pathology, Johns Hopkins School of Medicine, Baltimore, Maryland, USA.; 2 University of Maryland School of Medicine, Departments of Surgery and Microbiology and Immunology, Center for Vascular and Inflammatory Diseases, Baltimore, Maryland, USA.

## Abstract

T cell–mediated rejection (TCMR) develops after alloantigen-primed T cells migrate into an allograft to cause tissue damage. In contrast to antibody-mediated rejection, which creates lesions in the graft vasculature, injury to the graft vasculature is often limited during TCMR. In this issue of the *JCI*, Barba et al. investigated the mechanism by which the endothelium is spared from harm caused by graft-infiltrating CD8^+^ T cells. Endothelial cell protection was due to cell-extrinsic chemokine variations in the environment, rather than cell-intrinsic differences between endothelial and interstitial cells. The CXCL12 gradient in particular facilitated CD8^+^ T cell movement through the endothelial layer into the graft parenchyma. These findings suggest that targeting the CXCL12 pathway may prevent or alleviate TCMR.

## T cell–mediated rejection in kidney transplantation

Following transplantation of an allogeneic solid organ, recipient T and B cells can recognize donor HLA proteins and mediate alloimmunity. Both CD4^+^ and CD8^+^ T cells can respond to allogeneic HLA proteins, either through recognition of intact donor HLA proteins on graft tissue (i.e., direct pathway), or through peptides derived from polymorphic HLA proteins that are presented by recipient HLA proteins (i.e., indirect pathway) (1, 2). T cells primarily encounter alloantigens via antigen-presenting cells (APCs) in the secondary lymphoid organs, where they are activated before trafficking into graft tissue to trigger T cell–mediated rejection (TCMR) (3, 4).

Circulating T cells that enter graft tissue must extravasate through the donor graft vascular endothelium into the graft interstitium (4). This process is facilitated by a variety of signaling and adhesion receptors — including selectins, integrins, and chemokine receptors — expressed on activated T cells that enable binding to their cognate receptors or ligands on endothelium (5). However, the trafficking of T cells into vascularized allografts is also dependent on the presence of specific alloantigens (3). Paradoxically, although T cells interact with donor graft vascular endothelium in an antigen-specific manner, TCMR is characterized by damage to the tubular epithelium, whereas the endothelium is generally pathologically spared (6). This allograft endothelial immune evasion, or privilege, has been largely unexplained, but was thought to be due to cell-intrinsic mechanisms similar to those observed in other immune privileged anatomic sites. For example, immune privilege within the eye, central nervous system, and testes is established by low HLA class I and II expression, the presence of inhibitory surface receptors, and/or secretion of immunosuppressive cytokines. These cell-intrinsic mechanisms induce T cell anergy and prevent autoimmunity (7–10).

## Establishing endothelial immune privilege during TCMR

In this issue of the *JCI*, Barba et al. sought to better understand the mechanisms by which donor graft endothelium is spared from T cell–mediated damage (11). The authors first confirmed the histological paradox that TCMR does not encompass vascular lesions compared with antibody-mediated rejection (AMR). A single-cell RNA-Seq (scRNA-Seq) dataset of healthy donor kidneys initially established a lineage-specific gene set for each cell type. Microarray analysis of graft biopsies from AMR, TCMR, or no rejection indicated the proportion of endothelial and tubular epithelial lineage genes that were differentially expressed in each condition. While endothelial genes represented the vast majority (70.9%) of differentially expressed genes (DEGs) in AMR biopsies, they were only minimally represented (7.1%) in TCMR biopsies. In contrast, tubular epithelial genes were appreciably affected in TCMR versus AMR (92.9% vs. 29.1%, respectively). Additional scRNA-Seq data revealed that cell damage and stress pathways were upregulated within an endothelial cell cluster in AMR versus TCMR. Finally, the expression levels of multiple endothelial-mesenchymal transition markers, which indicate endothelial injury, were higher in AMR versus TCMR biopsies.

Having strengthened the finding that endothelium was relatively undamaged in TCMR relative to AMR, Barba and authors next used coculture experiments to determine whether endothelial cells had a cell-intrinsic mechanism of resistance to CD8^+^ T cells. Allogeneic HLA-A2–specific CD8^+^ T cells or unspecifically activated CD8^+^ T cells were cultured with either an HLA-A2^+^ glomerular endothelial or a tubular epithelial cell line as targets. HLA-A2–specific CD8^+^ T cells efficiently killed both endothelial and epithelial cells in an HLA-A2–dependent manner, while nonspecific CD8^+^ T cells failed to elicit a cytotoxic response. These results demonstrated that endothelial cells do not possess a cell-intrinsic resistance to CD8^+^ T cell–mediated cytotoxicity (11).

Next, the investigators determined whether the relative cytotoxic damage of epithelial versus endothelial tissue was reflected in the trafficking and/or localization of CD8^+^ T cells to specific tissue domains. Fluorescently labeled transgenic CD8^+^ T cells were adoptively transferred into C57Bl/6 mice that then received an OVA-expressing kidney allograft. The velocity and mean traveled distance of labeled T cells, as determined by 2-photon microscopy, were greater in the intravascular compartment than the extravascular compartment. A coefficient for arrest in cell movement, signifying the formation of cytotoxic immune synapses with allogeneic cellular targets, was higher in the extravascular compartment than the intravascular compartment. The investigators then devised a clever in silico model for assessing cell interactions and movement in human transplant biopsies. They found that the density of virtual CD8^+^ T cells was substantially higher in the tubular epithelial versus the vascular compartment. Together, these results provide evidence that, despite the absence of cell-intrinsic resistance to cytolysis by T cells, CD8^+^ T cells preferentially bypass the endothelial layer to interact more robustly with epithelial cells, which are the targets of cytotoxicity.

## Identification of a CXCL12 gradient in allograft tissue

Trafficking signals — specifically, a chemokine gradient within the tissue epithelial compartment — could be responsible for the movement of CD8^+^ T cells within the allograft. Barba and colleagues analyzed scRNA-Seq data from endothelial, interstitial, and tubular compartments and found that mRNA levels for *CXCL9*, *CCL2*, *CCL5* and, most strikingly, *CXCL12* were upregulated within the interstitium (11).

The authors set up an elegant killing assay to determine whether a CXCL12 gradient was sufficient to enable CD8^+^ T cells to bypass allogeneic endothelial cells. Under static conditions, meant to mimic the interstitial tissue environment, chemokine was present uniformly within the culture media. In contrast, dynamic chemotactic conditions, intended to mimic the transition from vasculature to interstitium, provided chemokine below the endothelial layer in a Transwell chamber. Notably, relative to static conditions, endothelial cells were less damaged by alloreactive CD8^+^ T cells migrating along a CXCL12 gradient under dynamic conditions. Similar results were obtained when tubular epithelial cells were cultured in the dynamic conditions, demonstrating the cell-extrinsic nature of this mechanism. In addition, the authors tested shear flow conditions to mimic CD8^+^ T cell trafficking through circulation in the context of inflammation. Endothelial cell damage was completely prevented in the presence of a CXCL12 gradient.

**Figure 1:**
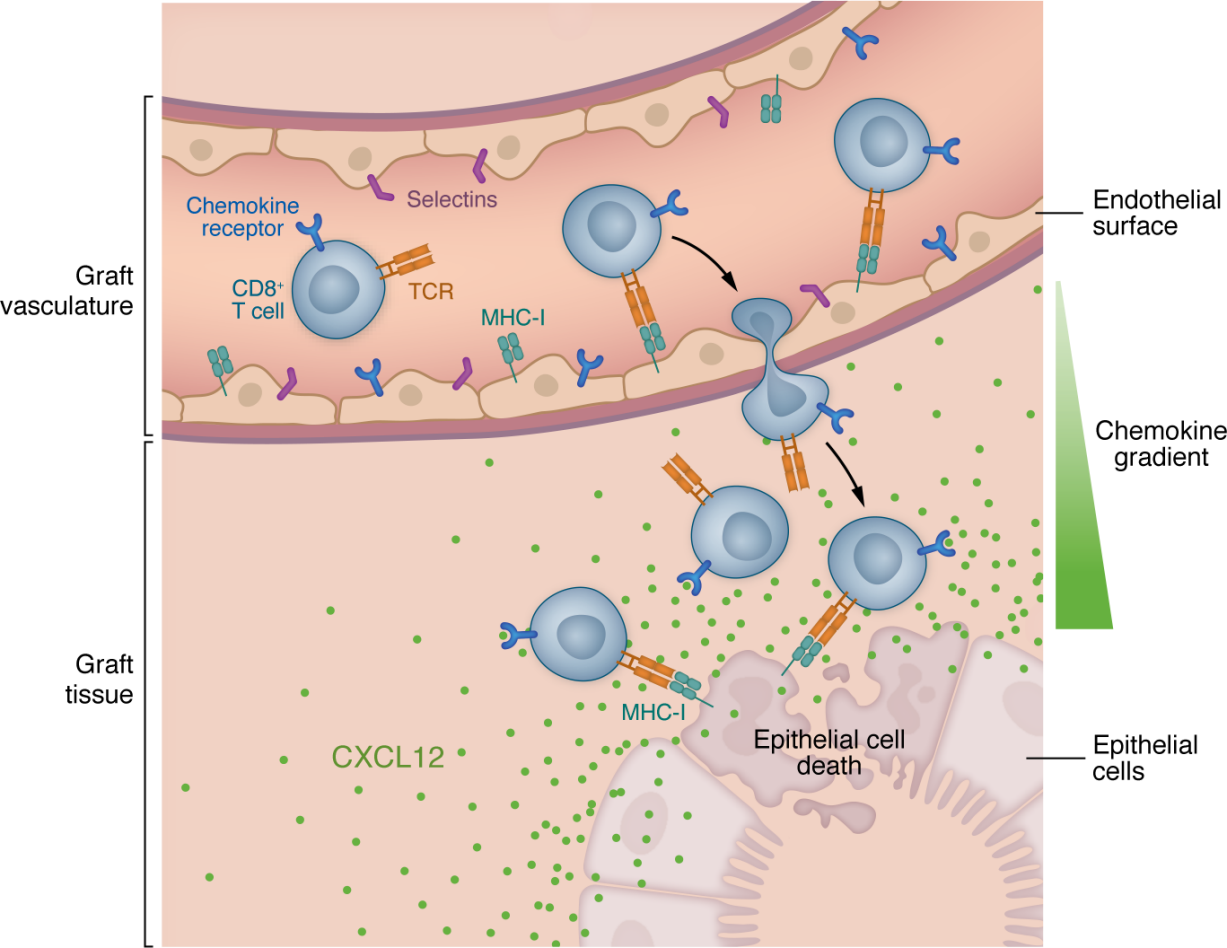
The CXCL12 gradient enables CD8^+^ T cells to bypass the allograft endothelial layer and mediate rejection. Alloantigen-specific CD8^+^ T cells are primed in the secondary lymphoid tissue and travel via the bloodstream to the allograft. In the graft vasculature, CD8^+^ T cells bind to cognate peptide-HLA complexes and adhesion receptors, including integrins, selectins, and chemokine receptors, and extravasate into the graft parenchyma. Despite the presence of cognate alloantigen, the migration of alloantigen-specific CD8^+^ T cells leaves the donor endothelial layer largely intact. Evidence from Barba et al. (11) suggests that alloantigen-expressing endothelial cells did not have a cell-intrinsic resistance to CD8^+^ T cell–mediated cytotoxicity. Rather, chemokine gradients within the allograft, such as CXCL12, promoted CD8^+^ T cell migration through the endothelial layer and into the graft interstitium, where graft epithelial cells are susceptible to cytotoxic injury.

Barba et al. (11) put forward the model that a CXCL12 chemokine gradient protects the endothelium from damage in most cases of TCMR (Figure 1). A corollary of this concept posits that pathologically severe TCMR, in which the endothelium becomes damaged, would coincide with a disrupted CXCL12 gradient. In a cohort of TCMR kidney biopsies with and without endothelialitis, computer assisted histomorphometry was used to assess the CXCL12 gradient. TCMR biopsies without endothelialitis exhibited a discernible chemokine gradient, while biopsies with endothelialitis had disruption of both the distribution and intensity of the gradient.

## Conclusions and future studies

Barba et al. (11) provide compelling evidence that a chemokine gradient can override antigen-specific killing by CD8^+^ T cells and thus establishes another form of immune privilege. Chemokine gradients were sufficient to prevent CD8^+^ T cell–mediated injury in multiple assays in vitro, and the disruption of a CXCL12 gradient in allograft biopsies corresponded with endothelial cell damage. This study suggests that chemokines play a dominant role in the trafficking of CD8^+^ T cells to targets within the allograft.

This chemokine-dependent mechanism is particularly intriguing, as it challenges the traditionally cell-intrinsic models of immune privilege, which rely on inhibitory receptors and the downregulation of MHC proteins to restrain immune responses. More specifically, these data modify our understanding of how CD8^+^ T cells balance antigen recognition versus chemokine signals.

In a vascularized kidney allograft model, Barba et al. (11) importantly demonstrated that graft-specific CD8^+^ T cells were preferentially found within the extravascular compartment relative to the graft vasculature. This finding extends prior seminal work by Lakkis and colleagues showing that graft-specific CD8^+^ T cell endothelial transmigration into vascularized allografts is dependent on cognate antigen expressed on either the endothelium or DCs (3). In both heart and kidney allografts, Walch et al. (3) showed that global blockade of chemokine signaling with pertussis toxin (PTX) was insufficient to prevent the endothelial transmigration of graft-specific, but not bystander, CD8^+^ T cells. By demonstrating that the chemokine gradient protects the vascular endothelium from cytotoxic damage in vivo, Barba et al. (11) provide a deeper mechanistic understanding of the role of chemokine signaling in the migration of CD8^+^ T cells into allografts. Barba et al. (11) suggest that disruption of chemokine signaling on CD8^+^ T cells in vivo, either through PTX or genetic deficiency, would remove endothelial cell privilege and result in cytotoxicity, although this remains to be directly shown.

While this work demonstrates that CXCL12 is sufficient to enable this form of immune privilege, future studies are needed to understand whether additional chemokine pathways contribute to this mechanism. Finally, the findings elevate the importance of therapeutic blocking of the CXCR4/CXCL12 pathway to prevent or alleviate TCMR.

## Version 1 07/15/2025

Electronic publication
